# Autoimmune nodopathy associated with Sjögren’s disease and nephrotic syndrome: a case report and literature review

**DOI:** 10.3389/fimmu.2026.1744214

**Published:** 2026-05-20

**Authors:** Xiaofeng Zhang, Di Niu, Ruizhi Chen, Haining Yu, Guoping Xing

**Affiliations:** 1 School of Clinical Medicine, Shandong Second Medical University, Weifang, Shandong, China; 2 Department of Neurology, Weifang People’s Hospital, Weifang, Shandong, China

**Keywords:** anti-contactin-1 antibody, autoimmune nodopathy, Sjögren’s disease, nephrotic syndrome, Guillain–Barré syndrome, chronic inflammatory demyelinating polyradiculoneuropathy

## Abstract

**Background:**

Anti-contactin-1 (CNTN1) antibody–positive autoimmune nodopathy (AN) is frequently reported in association with membranous nephropathy; however, the co-occurrence with both Sjögren’s disease and nephrotic syndrome represents an exceptionally rare comorbid phenotype. In this study, we explored the potential immunological links underlying this comorbidity and, in conjunction with a literature review, sought to identify diagnostic clues for AN.

**Case presentation:**

We report the case of a 45-year-old woman who presented with progressive numbness and weakness of all four limbs for 5 months, accompanied by xerostomia and bilateral lower limb edema. Nerve conduction studies demonstrated demyelinating features, and cerebrospinal fluid analysis revealed albuminocytologic dissociation. Serum testing was positive for anti-CNTN1 antibodies (titer 1:1000), along with nephrotic-range proteinuria (4.66 g/24 h) and positivity for anti-SSA52, anti-SSA60, and anti-SSB antibodies. The patient was ultimately diagnosed with anti-CNTN1 antibody–positive AN coexisting with Sjögren’s disease and nephrotic syndrome. Following intravenous methylprednisolone therapy, her neurological symptoms improved significantly.

**Conclusion:**

This case highlights that, within an immune context characterized by B-cell hyperactivation, anti-CNTN1 antibodies may mediate immune injury to both the peripheral nerves and kidneys by targeting shared antigens. Clinicians should recognize key diagnostic clues, including massive proteinuria, postural tremor, and poor response to intravenous immunoglobulin, to facilitate early identification of AN.

## 1 Introduction

Autoimmune nodopathy (AN) is a group of immune-mediated peripheral neuropathies characterized by autoantibodies targeting nodal and paranodal axoglial adhesion molecules. The target antigens can be broadly classified into paranodal proteins, including neurofascin-155 (NF155), contactin-1 (CNTN1), and contactin-associated protein 1, and nodal proteins, including neurofascin-186 and neurofascin-140 (1). Among these, anti-CNTN1 IgG4 antibody–positive AN is the second most common subtype after anti-NF155, accounting for approximately 0.7%–2.4% of chronic inflammatory demyelinating polyradiculoneuropathy (CIDP), and is more frequently observed in middle-aged and elderly men (2, 3). Sjögren’s disease is a systemic autoimmune disease characterized by B-cell hyperactivation, primarily affecting the salivary and lacrimal glands, but it may also involve the peripheral nervous system (4, 5). Diagnostic evaluation can be supported by minor salivar gland biopsy, serum autoantibodies, and the Schirmer test. Here, we report a rare case of coexisting AN, Sjögren’s disease, and nephrotic syndrome, aiming to clarify their potential immunological associations, avoid diagnostic pitfalls, and summarize key diagnostic clues.

## 2 Case presentation

### 2.1 Medical history and physical examination

A middle-aged woman with an unremarkable medical and family history, and no history of chronic medication use, was admitted to the Department of Neurology at our hospital with a five-month history of numbness and weakness in the limbs. The disease onset was subacute, with symptoms being persistent and progressively worsening. Three weeks prior to the onset, the patient reported a history of upper respiratory tract infection, for which no systematic medical treatment was sought. Subsequently, she developed symmetric numbness in the limbs, predominantly in the distal regions. In the upper extremities, the numbness initially manifested at the tips of the right middle and ring fingers, gradually progressing proximally to approximately 4 cm above both elbows. In the lower extremities, the numbness started at the soles of both feet and extended upward to the knees. Approximately two months after onset, the patient developed limb weakness, most notably distal to the wrists in the upper limbs and below the knees in the lower limbs. These symptoms were accompanied by xerostomia, reduced salivation, increased water intake, and recurrent oral and gingival ulcers. The patient had not received systematic treatment prior to admission. Approximately five months after onset, the aforementioned symptoms intensified significantly, accompanied by the development of gait instability.

Neurological Examination: The patient was alert and oriented, with fluent speech and intact higher cognitive functions. Cranial nerve examination was unremarkable. Muscle strength was grade V proximally and grade IV distally in both the upper and lower limbs. Pain and light touch sensations were reduced distal to the bilateral elbows and knees, and deep sensations, including vibration and position sense, were also impaired. Deep tendon reflexes, including the biceps, triceps, brachioradialis, patellar, and Achilles reflexes, were absent bilaterally. Finger-to-nose and heel-to-shin tests were mildly impaired. The Romberg test was unstable with both eyes open and closed, and tandem gait was unsteady. Bilateral pathological reflexes were absent, and meningeal signs were negative.

### 2.2 Ancillary examinations and diagnosis

**Figure 1:**
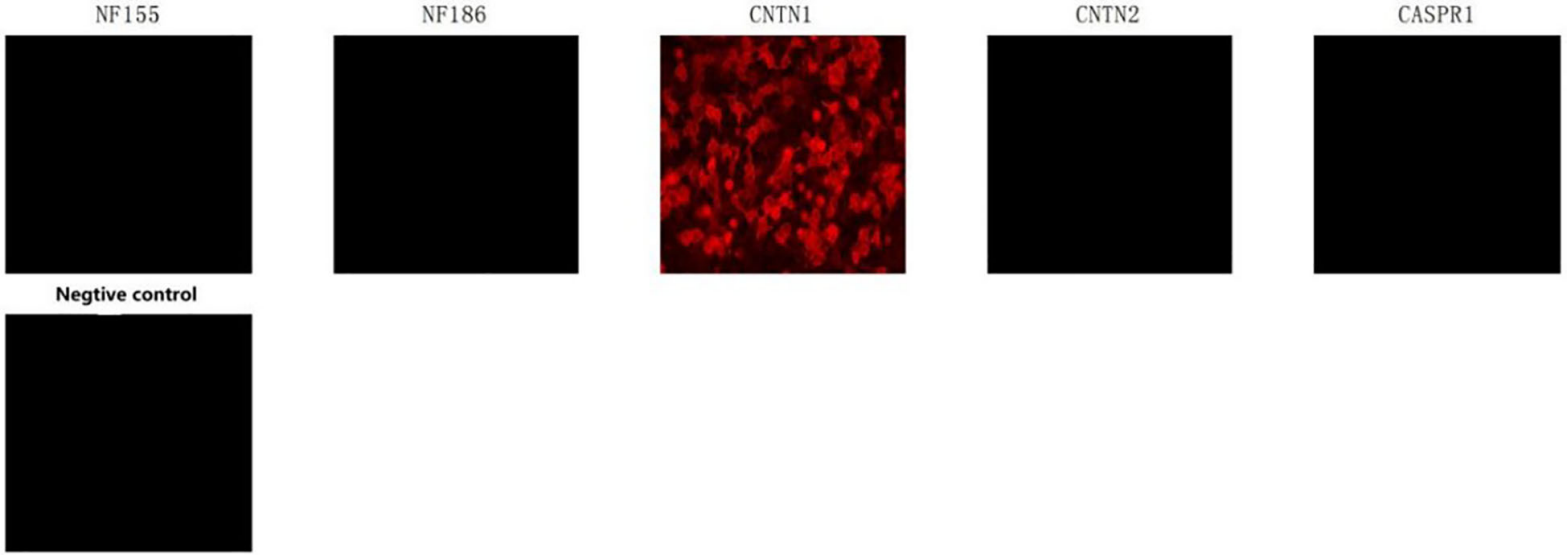
Anti-node of Ranvier-related antibody IgG (cell-based assay): Serum anti-CNTN1 IgG fluorescence staining was positive at 1:1000, whereas testing for other neurofascin and contactin-related autoantibodies-including anti-NF155, anti-NF186, anti-CASPR1, and anti-CASPR2 antibodies-yielded negative results.

**Figure 2:**
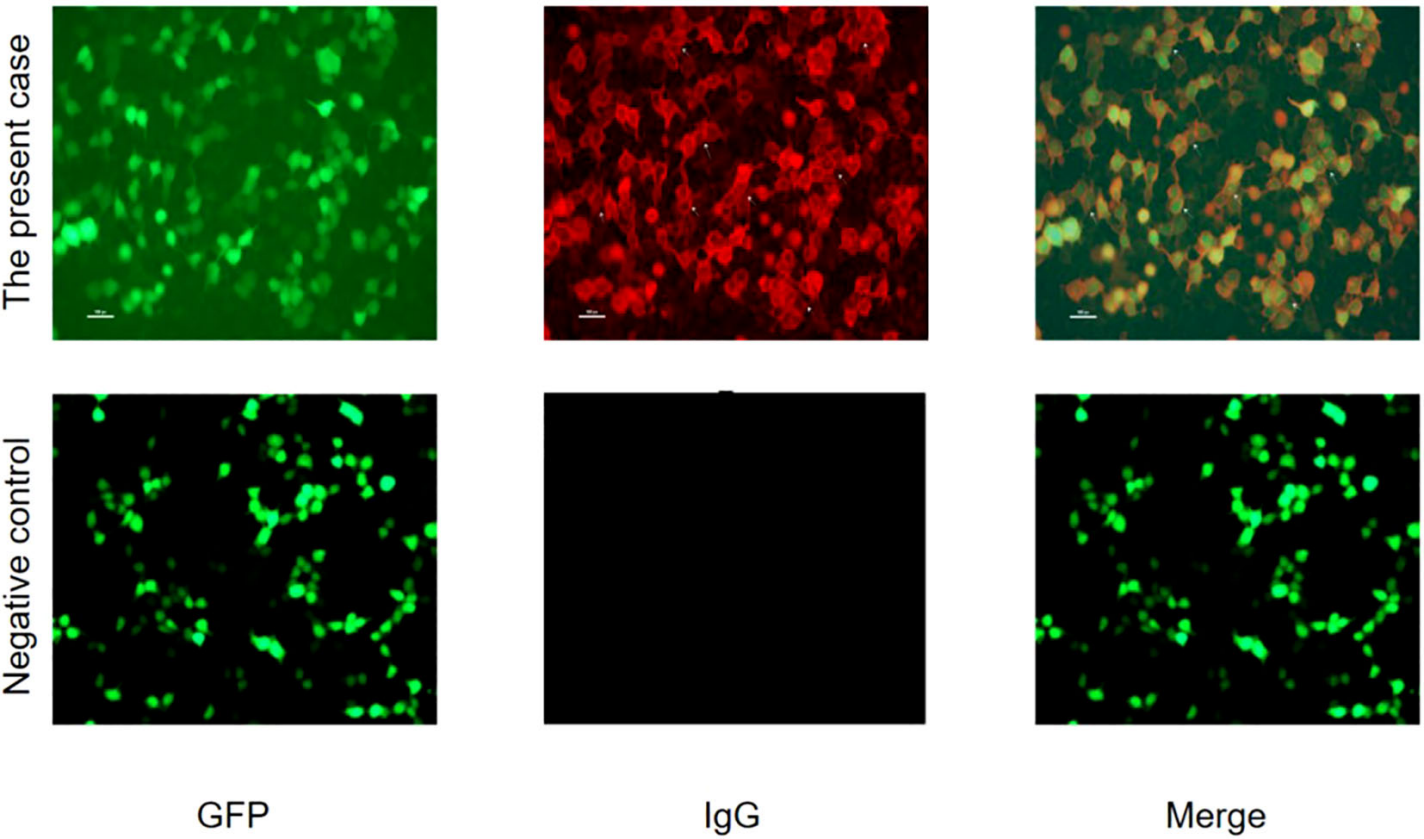
Fluorescence staining image of anti-CNTN1 antibody. The fluorescence images obtained through the anti-CNTN1 antibody transfection cell method reveal the following results: The GFP channel exhibits a positive signal (green fluorescence, suggesting that the transfected cells successfully express the GFP-tagged protein). The IgG antibody staining channel also shows a positive result (red fluorescence, indicating the specific expression of the target protein). In the Merge channel, co-localization of the two fluorescence signals can be observed (the yellow overlapping area).

Electromyography of the limbs revealed slowed motor nerve conduction velocities, prolonged distal latencies and F-wave latencies, and absent sensory nerve action potentials, consistent with a symmetric distal-predominant demyelinating peripheral neuropathy. Cerebrospinal fluid analysis demonstrated albuminocytologic dissociation (protein: 2820.5 mg/L, reference range: 150–400 mg/L; cell count: 4/μL, reference range: 0–5/μL). Serum testing for nodal and paranodal antibodies showed a strongly positive anti-contactin-1 (CNTN1) antibody (titer 1:1000; reference range <1:10; Jinan KingMed Diagnostics Laboratory) (**Figures 1**, **2**), confirming anti-CNTN1 antibody–positive autoimmune nodopathy (AN). In addition, the patient tested positive for anti-nuclear antibodies (speckled pattern, 1:320; reference range <1:100), anti-SSA52 antibodies (2.32; reference range 0–1), anti-SSA60 antibodies (2.53; reference range 0–1), and anti-SSB antibodies (2.26; reference range 0–1). The unstimulated whole salivary flow rate was 0.02 mL/min (reference range >0.1 mL/min). The patient had a history of xerostomia and xerophthalmia lasting more than three months. Based on the 2016 classification criteria of the American College of Rheumatology/European League Against Rheumatism, a diagnosis of Sjögren’s disease was established (6). Laboratory evaluation further revealed nephrotic-range proteinuria (24-hour urinary protein excretion: 4.66 g/day; reference range <0.15 g/day), hypoalbuminemia (serum albumin: 23.5 g/L; reference range 40–55 g/L), and hyperlipidemia (total cholesterol: 6.5 mmol/L, reference range 2.9–5.69 mmol/L; triglycerides: 2.64 mmol/L, reference range 0.4–2.25 mmol/L; low-density lipoprotein cholesterol: 4.80 mmol/L, reference range 1.5–4.3 mmol/L). In combination with the presence of bilateral pitting edema of the lower limbs, these findings met the diagnostic criteria for nephrotic syndrome. Serum IgG levels (29.6 g/L; reference range 8.6–17.4 g/L), the proportion of CD19^+^ B lymphocytes (31.7%; reference range 5–22%), and their absolute count (862/μL; reference range 80–616/μL) were all markedly elevated, suggesting an immune background characterized by B-cell hyperactivation. Meanwhile, negative results for anti-ganglioside antibodies, anti-neutrophil cytoplasmic antibodies, anti-Sm antibodies, anti-dsDNA antibodies, complement C3, complement C4, vitamin B12, and tumor screening (chest CT and abdominal ultrasonography) helped preliminarily exclude other conditions, including Guillain–Barré syndrome, Systemic Lupus Erythematosus, and paraneoplastic syndromes (**Table 1**).

**Table 1:** Summary of key laboratory findings and their diagnostic implications.

Category of findings	Test item	Result	Diagnostic implication
Positive findings	Anti-CNTN1 antibodies	Positive	Confirms anti-CNTN1 antibody–positive autoimmune nodopathy
	Anti-SSA/SSB antibodies	Positive	Supports the diagnosis of Sjögren’s disease
	24-hour urinary protein excretion	4.66 g/day	Supports the diagnosis of nephrotic syndrome
	Cerebrospinal fluid protein	2820.5 mg/L	Supports autoimmune demyelinating neuropathy
Negative findings	Cerebrospinal fluid cell count	4/μL	Helps exclude infectious peripheral neuropathy
	Anti-ganglioside antibodies	Negative	Helps exclude Guillain–Barré syndrome
	Anti-Sm antibodies, anti-dsDNA antibodies, complement C3/C4	Negative	Helps exclude systemic lupus erythematosus
	Anti-neutrophil cytoplasmic antibodies	Negative	Helps exclude vasculitis
	Tumor screening (chest CT / abdominal ultrasonography)	Negative	Helps exclude paraneoplastic syndrome

### 2.3 Diagnostic challenges and differential diagnosis

The clinical phenotype of AN closely resembles that of inflammatory demyelinating neuropathies, making it prone to diagnostic pitfalls. In this case, the patient presented with a subacute onset and a preceding infection, which initially made misdiagnosis as Guillain–Barré syndrome (GBS) highly likely. However, the disease course continued to progress, exhibiting features consistent with acute-onset chronic inflammatory demyelinating polyradiculoneuropathy (CIDP). Given the presence of xerostomia and proteinuria, along with positive anti-nuclear antibodies and anti-SSA and anti-SSB antibodies, neurological involvement associated with Sjögren’s disease or systemic lupus erythematosus also needed to be considered (1, 7–9) (**Table 2**).

**Table 2:** Differential diagnosis of GBS, CIDP, SLE, and Sjögren’s disease–associated peripheral neuropathy.

Diagnostic items	GBS (7)	CIDP (1)	Systemic lupus erythematosus (8)	Sjögren’s disease–associated peripheral neuropathy (9)
Main clinical features	Acute onset (reaching peak within 4 weeks); symmetric limb weakness with distal sensory impairment; decreased or absent tendon reflexes.	Progressive or relapsing limb weakness or sensory impairment lasting >8 weeks; symmetric involvement of both proximal and distal limbs.	Malar rash, discoid lupus, fever, oral ulcers, alopecia, arthritis, serositis, proteinuria, seizures and psychosis.	Distal symmetric numbness and weakness; non–length-dependent sensory loss and sensory ataxia; distal burning pain, paresthesia, and autonomic dysfunction.
Serological and cerebrospinal fluid examinations	Albuminocytologic dissociation; anti-ganglioside antibodies may be positive.	Albuminocytologic dissociation	ANA positive; anti-dsDNA and anti-Sm antibodies positive; decreased complement C3 and C4 levels.	Anti-SSA antibodies positive; some cases also positive for anti-SSB antibodies.
Electrophysiological features	Mixed neuropathic features, including conduction block, prolonged distal latencies, and axonal injury.	Demyelinating electrophysiological features, including slowed conduction velocity, temporal dispersion, conduction block, and prolonged F-wave latencies.	NA	Predominantly axonal injury
Treatment response	IVIg or plasma exchange is effective; glucocorticoids are usually ineffective.	IVIg, glucocorticoids, and plasma exchange are all first-line therapies.	Responsive to glucocorticoids and immunosuppressants.	IVIg and glucocorticoids are effective; in cases associated with vasculitis, rituximab or cyclophosphamide may be required.
Basis for exclusion in this case	The disease course continued to progress over a period of 5 months.	Subacute onset with positive anti-CNTN1 antibodies.	Negative anti-Sm and anti-dsDNA antibodies; complement levels within the normal range; no typical clinical manifestations of SLE.	Demyelinating electrophysiological features with confirmed anti-CNTN1 antibody positivity.

### 2.4 Treatment and outcome

Before the results of nodal/paranodal antibody testing were available, the patient was initially treated according to the therapeutic approach for CIDP. Considering economic factors and the fact that both glucocorticoids and intravenous immunoglobulin (IVIg) are first-line treatments, intravenous methylprednisolone (40 mg/day, administered with a 3-day tapering regimen) was initiated. The patient’s symptoms gradually improved, and this regimen was subsequently maintained. At the 6-month follow-up after discharge, the patient reported that she had not received any maintenance therapy. Xerostomia persisted, while neurological symptoms had markedly improved, with only mild residual weakness in the lower limbs.

## 3 Discussion

The clinical features of anti-contactin-1 (CNTN1) antibody–positive autoimmune nodopathy (AN) are highly consistent with the neurological manifestations observed in this case. These features include: (1) an acute or subacute onset with relatively rapid progression, often resembling Guillain–Barré syndrome in the early stage; (2) weakness predominantly affecting the distal limbs, frequently accompanied by sensory ataxia, with some patients developing prominent postural tremor, while others may present with predominantly sensory involvement; and (3) possible cranial nerve involvement and neuropathic pain. Among these manifestations, the pattern of onset and the presence of characteristic tremor represent key features that help distinguish AN from chronic inflammatory demyelinating polyradiculoneuropathy (CIDP), thereby providing important clues for early clinical recognition. This case illustrates a rare multi-target autoimmune axis. The immunological links among the three affected systems may be explained by integrating three potential mechanisms: molecular mimicry, shared antigens, and epitope spreading. Molecular mimicry may serve as the initiating trigger of disease. The patient had a clear history of respiratory tract infection three weeks before symptom onset, suggesting that exogenous pathogenic components may share structural similarities with human CNTN1 protein, thereby initiating a cross-reactive immune response. Although direct homology evidence for CNTN1-related molecular mimicry is currently lacking, the temporal association supports the classical mechanism of infection-triggered autoimmunity (10). Shared antigens may provide a pathological basis linking the involvement of different organs. Previous studies have demonstrated that CNTN1 is expressed not only in the paranodal regions of peripheral nerves but also in glomerular podocytes, where it can co-localize with IgG4 within glomerular immune complexes (11, 12). Therefore, anti-CNTN1 antibodies may simultaneously target both neural and renal tissues. In addition, epitope spreading may play a critical role during disease progression. The initial antigen-driven immune response may gradually broaden the spectrum of target antigens through intra- or intermolecular epitope spreading, thereby amplifying humoral immune responses and promoting disease progression. This mechanism has been well documented in antibody-mediated diseases such as membranous nephropathy (MN) and is closely associated with the expansion of antibody repertoires, increased antibody levels, and poorer clinical outcomes (13–15). In an autoimmune context characterized by abnormal B-cell activation, this process may further promote the production of multiple autoantibodies and contribute to multisystem involvement (16).

In recent years, increasing attention has been paid to the association between anti-CNTN1 antibody–related AN and MN. Primary MN is mediated by autoantibodies targeting podocyte antigens, ultimately leading to immune complex deposition on the epithelial side of the glomerular capillary wall. Although serum anti-phospholipase A2 receptor (PLA2R) and anti-thrombospondin type-1 domain–containing 7A (THSD7A) antibodies account for approximately 50%–80% and 5%–10% of cases, respectively (17), the pathogenic antigens in a subset of patients remain unidentified. With the advancement of research, CNTN1 has been identified as a novel pathogenic antigen in MN (18). In the present case, comprehensive screening for the above target antigens was not performed. However, based on the previously described shared antigen mechanism and the synchronous involvement of the peripheral nervous system and kidneys in this patient, our findings support the hypothesis that the nephrotic syndrome was caused by anti-CNTN1 antibody–mediated autoimmune glomerular injury, which is pathophysiologically highly consistent with the phenotype of CNTN1-related MN. An analysis by Ceyda et al. (19) of 39 patients with anti-CNTN1–positive AN demonstrated that renal pathology in all cases showed MN, further supporting the role of CNTN1 as a novel target antigen in MN (11). In addition, renal involvement has also been reported in patients with AN associated with antibodies against neurofascin-155 (NF155), neurofascin-140, and neurofascin-186, with pathological findings consistently demonstrating focal segmental glomerulosclerosis (19, 20). Studies have shown that patients with AN frequently present with varying degrees of proteinuria, a feature that is relatively uncommon in CIDP (21). Therefore, proteinuria may serve as a simple and effective clinical indicator that provides an important clue for the early recognition and diagnosis of AN.

Around 2010, antibodies associated with AN were first identified. However, it was not until 2021 that the European Academy of Neurology formally recognized AN as a distinct disease entity (1, 22). Prior to this recognition, these antibodies had not been incorporated into the diagnostic framework of inflammatory neuropathies, and clinicians might therefore have overlooked testing for them, potentially leading to misdiagnosis. To explore this issue, we conducted a literature search on PubMed using the MeSH terms “chronic inflammatory demyelinating polyradiculoneuropathy” and “Sjögren’s syndrome,” followed by secondary screening based on the core clinical features of anti-CNTN1 antibody–positive AN. After excluding cases with negative nodal/paranodal antibodies or those already diagnosed with AN, five cases highly suggestive of previously unrecognized AN were ultimately included (23–27) (**Table 3**). The selected cases closely matched the diagnostic clues identified in this study, providing supporting evidence for the hypothesis that AN is frequently misdiagnosed as CIDP.

**Table 3:** Reported cases highly suggestive of previously unrecognized autoimmune nodopathy.

Author and year	Gender	Age of onset	Mode of onset	Core clinical phenotype	Key electrophysiological features	Response to IVIg	Response to steroids /immunosuppressants	Nodal/paranodal antibody testing	Serological clues	Proteinuria	Key clues suggesting undiagnosed AN
Raquel (23), 2020	M	71	Chronic	Symmetric sensorimotor deficits in the limbs with progressive worsening	Absent distal sensory nerve action potentials in the limbs with conduction block in multiple nerves	Initially effective, with complete loss of efficacy later	Rituximab effective; long-term immunosuppression maintained remission	Negative (considered a false negative after 3 years of immunotherapy)	Anti-SSA/SSB antibodies positive	unknown	Loss of efficacy of IVIg in the later stage, with a good response to rituximab
Nimish (24), 2020	M	56	Subacute	Numbness and weakness in the limbs with sensory ataxia	Bilateral tibial nerve compound muscle action potentials were absent, with slowed conduction velocities in multiple nerves, prolonged distal latencies, absent sensory nerve action potentials, and prolonged F-wave latencies.	Completely ineffective	Neurological and renal manifestations improved simultaneously after cyclophosphamide therapy.	Not performed	Anti-SSA/SSB antibodies positive, anti-nuclear antibody positive	Nephrotic-range proteinuria	Complete resistance to IVIg, elevated IgG4, nephrotic-range proteinuria, and synchronous involvement of the peripheral nerves and kidneys
Landete (25), 1998	F	28	Subacute	sensory ataxia	Absent sensory nerve action potentials	Not used	Marked symptom improvement after methylprednisolone pulse therapy	Not performed	anti-nuclear antibody positive	Mild albuminuria	Subacute onset with a disease course of approximately 10 weeks, accompanied by mild albuminuria
Andrea (26), 2009	F	73	Subacute	Polyneuropathy predominantly characterized by distal limb weakness	Prolonged F-wave latencies in the bilateral ulnar and median nerves, with no response to stimulation of the peroneal and tibial nerves	Not used	Marked symptom improvement after prednisone therapy	Not performed	Anti-SSA/SSB antibodies positive, anti-nuclear antibody positive	unknown	Subacute onset with a disease course of approximately 3 months
Bregante (27), 2013	F	29	Subacute	Symmetric proximal and distal limb weakness with sensory impairment	Prolonged distal motor latencies	Completely ineffective	Glucocorticoid therapy induced remission, but long-term maintenance required intensified immunosuppressive therapy.	Not performed	Anti-SSA/SSB antibodies positive, anti-nuclear antibody positive	unknown	Subacute onset, complete resistance to IVIg, and steroid responsiveness

Differences in therapeutic response represent an important clinical clue for distinguishing AN from CIDP. Among the five cases summarized in this study, all three patients who received IVIg therapy showed either no response or only transient partial improvement followed by rapid loss of efficacy. This contrasts markedly with the typically high responsiveness of CIDP to IVIg. Notably, the case reported by Nimish et al. (24) was particularly illustrative: after IVIg treatment proved ineffective, the addition of cyclophosphamide resulted in concurrent improvement of both neurological symptoms and renal involvement. The limited therapeutic response of AN patients to IVIg is largely attributable to the unique structural properties of IgG4 antibodies. Their characteristic Fab-arm exchange allows half-molecule exchange *in vivo*, generating bispecific antibodies that functionally behave as monovalent molecules. Consequently, they cannot effectively cross-link antigens or form large immune complexes (28, 29). In addition, the Fc region of IgG4 antibodies has a conformation that confers relatively weak binding affinity to Fcγ receptors, particularly FcγRII and FcγRIII (28, 29). Since one of the principal pharmacological mechanisms of IVIg is to competitively saturate Fc receptors through its Fc fragments and thereby block the effector functions of pathogenic antibodies (30), the low affinity of IgG4 antibodies for these receptors significantly weakens the immunomodulatory effect of IVIg. Furthermore, patients with anti-CNTN1 antibody–positive AN may experience a shift in the dominant antibody subclass from IgG1 or IgG3 to IgG4 during disease progression (2, 31). Therefore, negative IgG4 antibody results should not preclude further diagnostic evaluation for this condition in clinical practice. This subclass switching may also explain why some patients initially respond to IVIg but subsequently lose therapeutic efficacy.

The treatment strategy in this case was individualized and based on evidence regarding anti-CNTN1 IgG4 antibody–positive AN. Recent classification studies have clarified that AN associated with anti-CNTN1 IgG4 antibodies belongs to the spectrum of IgG4 antibody–mediated autoimmune diseases (IgG4-AIDs). These conditions are characterized by pathogenic mechanisms primarily involving IgG4-mediated disruption of protein–protein interactions, rather than classical complement activation or Fc receptor–dependent inflammatory injury (32, 33). For IgG4-AIDs, glucocorticoids themselves are considered a standard first-line therapy (29). In addition, a study by Yumako et al. (2) demonstrated that approximately 73% of patients with anti-CNTN1 antibody–positive AN respond well to corticosteroid therapy, suggesting that this antibody may serve as a biomarker to guide therapeutic decision-making. For patients with inadequate responses to glucocorticoids, rituximab exerts therapeutic effects by targeting and depleting CD20-positive short-lived plasma cells, thereby reducing IgG4 antibody production. In patients with AN associated with antibodies against CNTN1, NF155, or contactin-associated protein 1 (CASPR1), rituximab has been reported to achieve clinical remission rates exceeding 80% (34), and it may provide greater benefit than glucocorticoid therapy in patients with concomitant membranous nephropathy (35). Previous studies have also suggested that long-term rituximab-mediated immunosuppressive therapy may reduce disease recurrence by depleting B-cell precursors and suppressing germinal center responses. This process is accompanied by decreased levels of key survival factors, including B-cell activating factor, a proliferation-inducing ligand, and interleukin-6, thereby disrupting the survival niches of plasma cells in the bone marrow and inflamed tissues and ultimately leading to a marked reduction in antibody-secreting cells (36, 37). However, considering the relatively high treatment cost and the potential risk of serious infections, the patient declined rituximab as a sequential first-line therapy after achieving symptomatic improvement with glucocorticoids. In the clinical management of AN, mycophenolate mofetil (MMF) is often regarded as an important second-line or adjunctive immunosuppressive therapy, as it can help maintain clinical remission while reducing glucocorticoid exposure (38, 39). After being fully informed of the potential adverse effects of MMF—including chronic diarrhea, abdominal pain, and the risk of bone marrow suppression—as well as the need for frequent outpatient follow-up and laboratory monitoring, the patient ultimately declined sequential MMF therapy based on considerations of treatment safety and convenience.

Electromyography in all five included cases demonstrated demyelinating electrophysiological features, including prolonged distal motor latencies, reduced conduction velocities, or absent sensory nerve action potentials, which were highly consistent with the electrophysiological findings observed in the present case. This pattern differs from the electrophysiological characteristics of peripheral neuropathy associated with Sjögren’s disease, which is typically characterized by predominant axonal injury (40). Based solely on electrophysiological examination, it is often difficult to distinguish anti-CNTN1 antibody–positive AN from CIDP. However, their pathological features differ substantially. In contrast to CIDP, anti-CNTN1 antibody–positive AN does not exhibit the characteristic onion-bulb formations caused by repeated demyelination and Schwann cell proliferation and repair (41). The underlying mechanism of this difference lies in the unique axoglial disjunction associated with anti-CNTN1 antibody–positive AN. Under physiological conditions, CNTN1 and CASPR1 interact with Schwann cell NF155 to form an axoglial adhesion complex that maintains the structural stability of the paranodal region. *In vitro* cell aggregation assays and animal studies have demonstrated that anti-CNTN1 antibodies can directly inhibit the interaction between CNTN1/CASPR1 and NF155, thereby disrupting this complex (42). Further *in vivo* studies have shown that these antibodies lead to the loss of CNTN1, CASPR1, and NF155 at the paranodal region, resulting in disintegration of the paranodal structure (12). Pathological observations have also confirmed the detachment of Schwann cell paranodal loops from the axonal membrane (43). Unlike classical inflammatory demyelination, this axoglial disjunction leads to functional disruption of the paranodal structure, which manifests electrophysiologically as slowed conduction velocities or conduction block (12). It is noteworthy that in the case reported by Raquel et al. (23), nodal/paranodal antibodies were negative four years after disease onset. However, the patient had previously received long-term high-frequency IVIg therapy for three years. This serological conversion may have been influenced by methodological interference. High concentrations of exogenous IgG antibodies may produce masking effects or matrix interference in enzyme-linked immunosorbent assays or cell-based assays, thereby significantly reducing detection sensitivity and potentially leading to false-negative results for anti-CNTN1 antibodies (44, 45).

## 4 Limitations

This study has several limitations. First, due to financial constraints, the patient did not undergo renal biopsy, including light microscopy, immunofluorescence, and electron microscopy, nor was comprehensive serological screening for primary MN–related antibodies, such as anti-PLA2R and anti-THSD7A antibodies, performed. Renal biopsy is the gold standard for determining the pathological subtype of kidney disease and for directly demonstrating antibody deposition. Serological testing helps exclude common forms of primary MN. The absence of these examinations prevented us from confirming the diagnosis of MN and from definitively demonstrating that the nephrotic syndrome was caused by anti-CNTN1 antibody–mediated autoimmune glomerular injury. Second, although the patient’s clinical presentation and strongly positive anti-CNTN1 antibodies strongly suggested concurrent involvement of the nerve–kidney axis, the conclusion that a shared antigen, namely CNTN1 expressed in both paranodal regions of peripheral nerves and glomerular podocytes, was involved remains inferential because tissue-level immunofluorescence localization was not available. Third, this report is based on the observation and analysis of a single case, and therefore the conclusions cannot be generalized to all patients with AN accompanied by multisystem involvement. Future multicenter prospective studies including larger cohorts are required to further clarify the potential association between anti-CNTN1 antibodies and systemic autoimmune diseases.

## 5 Conclusion

This case further supports a potential immunological association between anti-CNTN1 antibody–positive autoimmune nodopathy, renal injury, and Sjögren’s disease. When evaluating patients with suspected chronic inflammatory demyelinating polyradiculoneuropathy or Guillain–Barré syndrome, clinicians should consider the possibility of AN if marked proteinuria, limited response to intravenous immunoglobulin, or characteristic postural tremor and sensory ataxia are observed. In patients with peripheral neuropathy occurring in the context of B-cell hyperactivation, early screening for antibodies against paranodal proteins is essential to avoid misdiagnosis.

## Data Availability

The datasets presented in this article are not readily available because of ethical and privacy restrictions. Requests to access the datasets should be directed to the corresponding author.

## Glossary

AN: Autoimmune Nodopathy
MN: Membranous Nephropathy
CIDP: Chronic Inflammatory Demyelinating Polyradiculoneuropathy
GBS: Guillain–Barré
IgG4-AIDs: IgG4 Antibody–Mediated Autoimmune Diseases
MMF: Mycophenolate Mofetil
IVIg: Intravenous Immunoglobulin
CNTN1: Contactin-1
NF155: Neurofascin-155
CASPR1: Contactin-Associated Protein 1
PLA2R: Phospholipase A2 Receptor
THSD7A: Thrombospondin Type-1 Domain–Containing 7A
